# Identification and Categorization of Liver Toxicity Markers Induced by a Related Pair of Drugs

**DOI:** 10.3390/ijms12074609

**Published:** 2011-07-15

**Authors:** Ching-Wei Chang, Frederick A. Beland, Wade M. Hines, James C. Fuscoe, Tao Han, James J. Chen

**Affiliations:** 1 Division of Personalized Nutrition and Medicine, National Center for Toxicological Research, FDA, Jefferson, AR 72079, USA; E-Mail: ching-wei.chang@fda.hhs.gov; 2 Division of Biochemical Toxicology, National Center for Toxicological Research, FDA, Jefferson, AR 72079, USA; E-Mail: FrederickBeland@fda.hhs.gov; 3 BG Medicine, Inc. Waltham, MA 02451, USA; E-Mail: wade.hines@gmail.com; 4 Division of Systems Biology, National Center for Toxicological Research, FDA, Jefferson, AR 72079, USA; E-Mails: James.Fuscoe@fda.hhs.gov (J.C.F.); Tao.Han@fda.hhs.gov (T.H.);

**Keywords:** liver toxicity, biomarker, genomic, personalized medicine, population heterogeneity, entacapone, tolcapone

## Abstract

Drug-induced liver injury (DILI) is the primary adverse event that results in the withdrawal of drugs from the market and a frequent reason for the failure of drug candidates in the pre-clinical or clinical phases of drug development. This paper presents an approach for identifying potential liver toxicity genomic biomarkers from a liver toxicity biomarker study involving the paired compounds entacapone (“non-liver toxic drug”) and tolcapone (“hepatotoxic drug”). Molecular analysis of the rat liver and plasma samples, combined with statistical analysis, revealed many similarities and differences between the *in vivo* biochemical effects of the two drugs. Six hundred and ninety-five genes and 61 pathways were selected based on the classification scheme. Of the 61 pathways, 5 were specific to treatment with tolcapone. Two of the 12 animals in the tolcapone group were found to have high ALT, AST, or TBIL levels. The gene Vars2 (valyl-tRNA synthetase 2) was identified in both animals and the pathway to which it belongs, the aminoacyl-tRNA biosynthesis pathway, was one of the three most significant tolcapone-specific pathways identified.

## 1. Introduction

Many approved drugs are removed from the market after the post-marketing discovery of unexpected adverse events, such as liver or kidney toxicity that were not detected in extensive pre-clinical testing. The occurrence of drug-induced liver injury (DILI) [1] is the single most common reason for the Food and Drug Administration’s regulatory actions concerning drugs [2]. Hepatotoxicity accounted for about 27% of drugs withdrawn from the market in the period 1960–2002 and over 40% of clinical phase drug candidate terminations due to toxicity [3–8]. Beyond the direct cost in human suffering and healthcare expenditure, the cost of DILI to pharmaceutical companies has been substantial, with a cost of $2 billion over a 10 year period [9]. Clearly, there is a need to prevent DILI, either by preventing drugs that have a liability for causing liver injury from entering the marketplace or by close monitoring of patients who take drugs that carry such a liability, particularly if the drug is unique in its potential for therapeutic benefit.

The occurrence of DILI may be observed during the pre-clinical or clinical development of a drug, or after its approval and subsequent marketing. For example, in pre-clinical development, DILI may be observed where the animal species studied exhibits clear signs of liver toxicity, such as liver enzyme elevations, histopathological alterations, or both, at dosing regimens that are associated with the efficacy of the drug. In clinical phase I, II, or III trials, the subjects/patients may exhibit signs of liver toxicity, despite the absence of liver toxicity in animal species during preclinical development. Finally, after the drug is on the market, there may be incidences of DILI in the target patient population, despite the absence of signs of liver toxicity throughout the entire pre-clinical and clinical drug development process.

DILI that is recognized only after marketing a drug generally has an incidence too low to be detected in typical Phase III clinical trials. This type of liver toxicity, which has an incidence of 1 in 10,000 to 1 in 100,000, is referred to as “idiosyncratic”, indicating the key role of individual susceptibility in the generation of the liver injury. The factors that determine the susceptibility of certain subsets of patients toward a particular drug are both genetically determined as well as environmental, including secondary diseases, nutritional status, polymedication, cellular stress, or infections [10].

The occurrence of a small number of hepatotoxic events may result in withdrawal of a drug from the market, even though the drug may benefit the vast majority of those taking it without increased risk of liver injury. More knowledge of the pre-clinical and clinical signatures of idiosyncratic drug reactions could help to predict hepatotoxicity. Increased efforts to identify patient-specific risk factors and response factors will improve the predictability of idiosyncratic hepatotoxicity in drug development as well as help to define patient subsets at risk and assist in candidate selection in new drug development [10]. Sensitive subpopulations of patients are not presently identifiable in advance using conventional diagnostic criteria. However, if sensitive subpopulations could be characterized in advance, then drugs might be approved selectively for the majority of patients who do not belong to these sensitive subpopulations. This could greatly reduce the risk of idiosyncratic hepatotoxicity and allow drugs that are beneficial to large segments of the population to remain on the market.

In this study, we hypothesized that despite the absence of conventional indicators of liver toxicity in pre-clinical studies, there exist biochemical signals (molecular biomarkers) in liver or body fluids that can be used to distinguish between a drug candidate that has the potential to cause DILI in susceptible patients and drugs that do not have this potential [11]. A molecular systems approach to DILI has the potential to provide pre-clinical molecular biomarkers that indicate the potential for a drug candidate to cause idiosyncratic liver injury in the clinic when no conventional signs, such as liver enzyme elevations or histopathological changes, are observed in animal studies. This paper presents an approach for identifying potential liver toxicity genomic biomarkers from a pre-clinical liver toxicity biomarker study.

The Liver Toxicity Biomarkers Study (LTBS) [11] presents an innovative approach for identifying a set of biomarkers to predict the probability of a compound causing hepatocellular injury in a subset of patients, despite the absence of conventional pre-clinical histopathology or clinical chemistry indicators of liver toxicity. The LTBS is a collaborative pre-clinical research effort in molecular systems toxicology between the U.S. Food and Drug Administration’s National Center for Toxicological Research and BG Medicine, Inc., and is supported by seven pharmaceutical companies and three technology providers. The key feature of the research strategy is a comprehensive molecular systems analysis of differences between the tissue and/or body fluid biochemical responses of rats to pairs of drugs, one a “non-liver toxic drug” and the other a “hepatotoxic drug”. In Phase I of the LTBS, the drugs entacapone and tolcapone were studied; both compounds were reported to be free of liver toxicity in pre-clinical studies. Both tolcapone and entacapone are potent inhibitors of catechol-*O*-methyl transferase and used for the treatment of Parkinson’s disease. Tolcapone was withdrawn from the market because of lethal hepatotoxicity, but has been reintroduced with a “black box” warning label since liver toxicity could be detected and reversed. No cases of life-threatening hepatotoxicity have been reported for patients taking entacapone.

The Phase I experiment was a 28-day oral rat toxicity study with a vehicle and 3-dose-level design. Briefly, six-week-old male Sprague-Dawley rats (12 per group) were dosed daily by gavage for 28 days with either entacapone (30, 110, or 400 mg per kg body weight) or tolcapone (15, 55, or 200 mg per kg body weight). Control rats were treated with the vehicle (0.5% methyl cellulose). Additional details are provided in the Section 3.1 and in McBurney *et al.* [11].

Neither tolcapone nor entacapone had any effect upon body weights, liver weights, or liver-to-body weight ratio. After 28 days of dosing, the animals were euthanized; blood and liver were collected for clinical chemistry, histopathology, proteomic, metabolomic, and/or microarray gene expression analyses. The bio-analytical platforms for the proteomic, metabolomic, and microarray experiments were performed only on the samples from the male rats. Detailed descriptions of experimental design and platforms of the LTBS are given in McBurney *et al.* [11]. The current paper focuses on the analysis of the liver tissue global gene expression data. The approach presented can also be applied to proteomic and metabolomic data.

## 2. Results and Discussion

### 2.1. Classification Scheme

The main goal of the LTBS is to provide lists of markers grouped into categories to distinguish among vehicle, entacapone, and tolcapone, and between entacapone and tolcapone. We propose four categories of markers based on the outcomes of statistical tests.

Tolcapone (t) markers show statistically significant differences in T-V (tolcapone *versus* vehicle) but not in E-V (entacapone *versus* vehicle) comparisons. If the genes also show statistically significant differences in T-E (tolcapone *versus* entacapone) comparisons, these genes are considered to be ideal tolcapone-specific markers (T). In addition to calculating the *p*-values, the direction of gene expression change between drug treatment and control was noted (*i.e*., gene expression was either higher or lower in the treated rat liver compared to control rat liver). Both drugs have the same directional effect on the marker’s expression (*i.e*., both up or both down) relative to the vehicle.

Entacapone (e) markers show statistically significant differences in E-V but not in T-V comparisons. The ideal entacapone-specific markers (E) are also statistically significant in the T-E comparison. Both drugs have the same direction of effect on the marker’s expression relative to the vehicle.

Common (c) markers show statistically significant differences in T-V and E-V comparisons and both drugs have the same directional effect from the vehicle. The ideal common-behavior markers (C) are not significant in the T-E comparison.

Divergent (d) markers show statistically significant differences in T-E comparisons, and both drugs differ from the vehicle and have an opposite directional effect (e.g., one up and one down) from the vehicle. The ideal divergent behavior markers (D) are also significant in the T-V and E-V comparisons.

**Figure 1:**
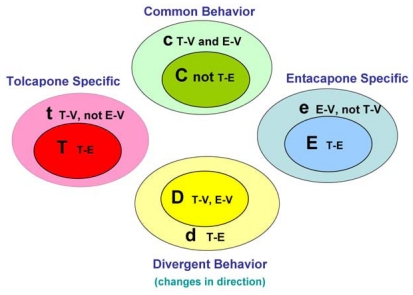
The ideal categories (C, T, E and D) and the primary categories (c, t, e, and d). The ideal category is a subset of the corresponding primary category.

**Figure 2:**
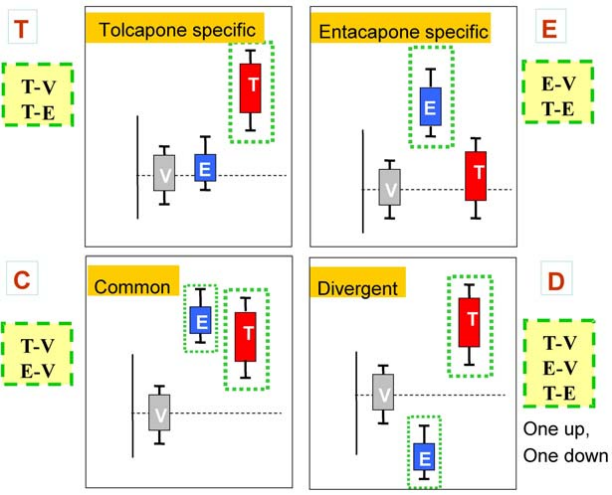
An illustration of the four ideal categorizations based on the statistical significance testing.

The above eight classification categories seek to capture the ideal markers from the full spectrum of possible responses. Figure 1 illustrates the four primary and four ideal categories. Figure 2 presents schematic representations of the four categories resulting from the three comparisons, T-V, E-V, and T-E.

The classification scheme described above can be applied directly using the vehicle and one dose group of each drug, such as the tolcapone high-dose and entacapone high-dose group. However, because of biological variability in the responses to the dose, the use of a particular dose to determine drug effects for all genes will be suboptimal. Therefore, an approach using the full range of dose-response data was used and is described below.

### 2.2. Clinical Chemistry Variables

Table 1 shows the means and standard errors in clinical chemistry parameters for the seven experimental groups, and the results of ANOVA and all pair-wise comparisons from the Tukey’s multiple range test. In drug and vehicle comparisons, there was only one statistically significant difference (BUN) between the entacapone high-dose and vehicle groups (*p* = 0.011). In tolcapone and entacapone comparisons, there was a statistically significance difference between the tolcapone high dose and entacapone low dose groups for ALT (*p* = 0.026) and AST (*p* = 0.044). Also, the high dose group of tolcapone showed differences from its low and medium dose groups in ALT; and the low dose group of tolcapone showed a difference from the low dose of entacapone in TBIL (*p* = 0.048).

**Figure 3:**
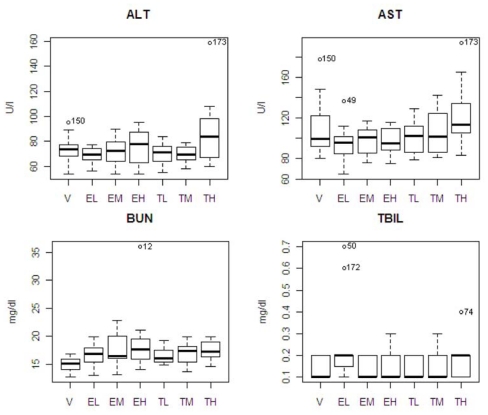
Box plots of the clinical chemistry variables that were statistically significant. The variable names of x-axis indicate vehicle (V), entacapone low dose (EL), entacapone medium dose (EM), entacapone high dose (EH), tolcapone low dose (TL), tolcapone medium dose (TM), and tolcapone high dose (TH). The IDs of the likely ‘outlying animals’ of treatment groups are indicated.

The pattern of chemistry parameter changes and dose levels were also studied. ALT showed an increase at only the highest dose. AST and TBIL showed an increasing pattern associated with dose level with tolcapone, but the difference was not significant. BUN showed increasing pattern associated with dose level with tolcapone and entacapone, but the differences were not significant. A box plot of the four statistically significant clinical chemistry variables is shown in Figure 3. One animal in the entacapone high dose group (animal #12) had an extremely high level of BUN based on IQR criterion [12]. If this animal was excluded from the analysis, then the difference between the entacapone high dose and vehicle groups was no longer statistically significant. Similarly, the statistically significant difference between the entacapone low-dose and tolcapone low-dose groups in TBIL appeared due to two animals in the entacapone low-dose group having extreme levels of TBIL. Two animals from tolcapone high dose group (animals #173 and #74) have high levels of ALT, AST, and TBIL. These animals will be discussed in detail below.

### 2.3. Microarray Genomic Biomarkers

Table 2 shows the number of classified markers identified by the significance testing approach for the 4 ideal marker categories (T, E, C, and D) and the 4 primary categories (t, e, c, and d). Six hundred ninety-five (695) genes were identified, of which 238 genes were classified as ideal markers, T, E, C, and D. Self-organizing map (SOM) clustering, with a 5 × 5 grid structure, was then applied to the 695 markers. Among the 695 genes, 472 genes were classified by SOM into the same t, e, c, and d categories a 68% concordance. Furthermore, 138 of the 238 primary marker genes were consistently classified into the same T, E, C, or D categories, a 58% concordance. The sensitivity and specificity of each of the markers were also calculated based on prediction results from logistic discrimination analysis. The range of average sensitivity and specificity are from 0.71–0.74 and 0.69–0.73 respectively (Table 2).

**Figure 4:**
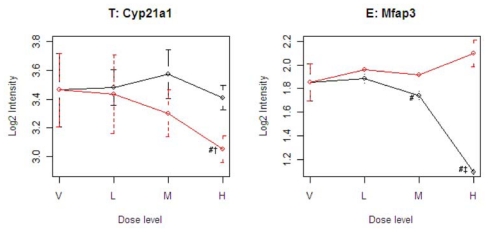

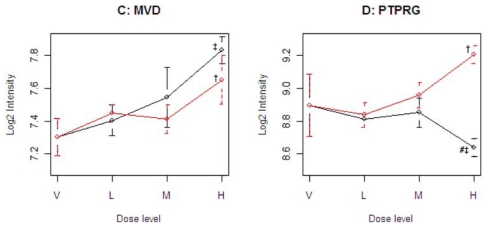
Gene expression patterns and standard errors of four representative genes for the four categories, T, E, C, and D. The variable names on the x-axis indicate vehicle (V), low dose (L), medium dose (M), and high dose (H). The black line is entacapone, and the red line is tolcapone. Some standard errors of Mfap3 are too small (<0.05) to display. The symbol # indicates a significant difference between tolcapone and entacapone. The symbol † indicates a significant difference between tolcapone and vehicle. The symbol ‡ indicates a significant difference between entacapone and vehicle.

Figure 4 illustrates four representative genes, Cyp21a1 (T), Mfap3 (E), MVD (C), and PTPRG (D), identified by the SOM for the four ideal categories. In Figure 4, the tolcapone-specific gene Cyp21a1 encodes a member of the cytochrome P450 superfamily of enzymes. This gene is also annotated in the C21-steroid hormone metabolism pathway. Since the C21-steroid is highly related to liver function [13], the difference implies that the two compounds affect the liver differently. The entacapone-specific gene Mfap3 (microfibrillar-associated protein-3) is annotated as a component of elastin-associated microfibrils. Microfibrils of diameter 10–12 nm are found either in association with elastin or independently and are an important component of the extracellular matrix of many tissues. The proteins composing the microfibrils are distinct from elastin. The largest and possibly the most important of these are the fibrillins, 350-kD glycoproteins that form integral parts of the microfibril structure [14]. The Common-Behavior gene MVD (mevalonate (diphospho) decarboxylase) is annotated with ATP binding, kinase activity, and catalysis of the diphosphomevalonate decarboxylase activity. In one of the early steps in the biosynthesis of cholesterol in the liver, MVD catalyzes the conversion of mevalonate pyrophosphate into isopentenyl pyrophosphate. The Divergent Behavior gene PTPRG (Receptor-type tyrosine-protein phosphatase gamma) is a member of the protein tyrosine phosphatase family involved in cell signaling. In conjunction with protein kinases, these enzymes control the state of phosphorylation of cell proteins and thereby provide an important mechanism for regulating cellular activity.

### 2.4. Pathway Analysis

Of the 193 candidate pathways identified to contain genes included in these analyses, 61 pathways included at least one categorized gene identified by the significance and SOM methods. Forty-eight of the 61 pathways showed significant differences among the three groups, tolcapone, entacapone, and vehicle. Eight of the 61 pathways which contained only T and/or t category genes were specific for tolcapone (Table 3). Five of the eight tested as significant at *p* ≤ 0.05 from the MANOVA test (Section 3.3). The three most significant pathways, galactose metabolism, cysteine metabolism, and aminoacylt-RNA biosynthesis, contain the three genes, Gck, Sult1c2, and Vars2, respectively; all three genes are in the Category T and were confirmed by the SOM. Vars2, is also related to the susceptible animals, and will be discussed further. It is noted that the identified pathways may not directly link to tolcapone metabolism, but rather reflect toxicity; however, these identified pathways and genes are good candidates for further research.

The galactose metabolism pathway is involved in the regulation and function of carbohydrate response element binding protein, ChREBP, in liver. Browning and Horton [15] have highlighted ChREBP as contributing to hepatic steatosis and nonalcoholic steatohepatitis. Glucose activates ChREBP by regulating the transition of ChREBP from the cytosol into the nucleus and by activating the binding of the transcription factor to DNA [16]. Glucose stimulates ChREBP to bind to an E-box motif in the promoter of liver-type pyruvate kinase (L-PK), a key regulatory enzyme in glycolysis. L-PK catalyzes the conversion of phosphoenolpyruvate to pyruvate, which enters the Krebs cycle to generate citrate, the principal source of acetyl-CoA used for fatty acid synthesis. Thus, activation of L-PK stimulates both glycolysis and lipogenesis, thereby facilitating the conversion of glucose to fatty acids under conditions of energy excess. Whether inactivation of ChREBP will attenuate the development of fatty livers in insulin-resistant states is currently under investigation by Browning and Horton [15].

The cysteine metabolism pathway is another significant pathway from the gene set analysis. The cysteine metabolism pathway has been investigated in relation to the increased severity of alcoholic liver injury in female verses male rats [17]. The categorized gene Sult1c2 in the pathway is also reported to be involved in ascorbic acid enhancing the liver tumor-promoting activity of kojic acid in rats [18].

### 2.4. Two Susceptible Animals

Table 1 shows statistically significant differences in ALT and AST between the tolcapone high dose and entacapone low dose groups. Figure 1 shows that the animal #173 had high ALT and AST levels, and #74 had a high TBIL level. If the two animals were excluded from the analysis, the tolcapone effects were no longer significant in the comparisons. This indicates that some of the measured clinical variables may be good indicators of liver toxicity in specific sensitive animals, but are poor indicators in normal healthy populations of animals.

The intensity levels for the expression of the tolcapone-specific genes were examined further for these two animals. For animal #173, which had high ALT and AST levels, six out of 120 T-specific genes were found to have higher levels of expression. The six genes included A1bg, G0s2, Kcnk3, Vars2, and two unknown probesets. For the animal #74, which had high level of TBIL, three genes were found. The three genes included Calcr, Vars2, and one unknown probeset.

**Figure 5:**
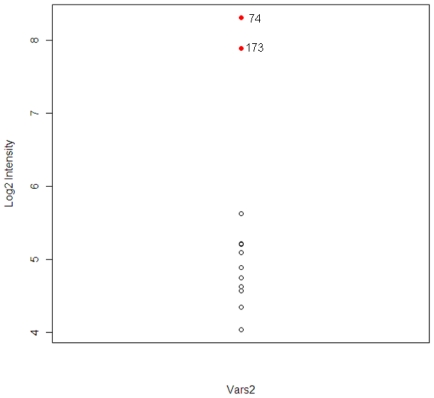
The expression levels of Vars2 for the 12 animals in tolcapone high dose group. The red circles indicate the animals #74 and #173.

The gene Vars2 (valyl-tRNA synthetase 2) was identified in both animals and the pathway to which it belongs, the aminoacyl-tRNA biosynthesis pathway, was one of the top three most significant tolcapone-specific pathways identified (Table 3). Vars2 is a member of the valyl-tRNA synthetase protein family. It is involved in the functions of nucleotide binding and tRNA aminoacylation for protein translation, and related to acetaminophen-induced liver disease [19]. The expression distribution of Vars2 for the 12 animals is shown in Figure 5. The two extreme observations are animal #173 and #74. The correlations between Vars2 and the chemistry parameters, which were used to identify the two animals, were calculated. The correlations are 0.78, 0.65, and 0.73 for ALT, AST, and TBIL respectively.

## 3. Experimental Section

### 3.1. Experimental Design

The experimental groups included a control group and 3 dose groups, with 12 male rats in each group. Treatment began when the animals were six weeks of age. Entacapone was administered by oral gavage once daily at doses of 30, 110, and 400 mg per kg body weight. Tolcapone was administered by oral gavage once daily at doses of 15, 55, and 200 mg per kg body weight. The dose levels were based on available efficacy and safety information for both drugs [20–22]. The dosing range encompassed efficacious dosing in rat and man (lowest doses) and highest doses tolerated without conventional indications of toxicity, with the middle doses set at an approximate geometric mean. Both drugs were administered as suspensions in 0.5% methyl cellulose. Control rats were treated with the vehicle. Food (NIH-31 pellets) and water were available *ab libitum*. The treatment continued for 28 consecutive dose days and the rats were sacrificed on day 29. After sacrifice, serum, plasma and liver samples were harvested and analyzed for the molecular profiling data. Additional details are provided in McBurney *et al.* [11].

Plasma clinical chemistry variables included aspartate aminotransferase, alanine aminotransferase, alkaline phosphatase, urea nitrogen, creatine kinase, lactate dehydrogenase, creatinine, glucose, total cholesterol, phospholipids, triglycerides, total protein, albumin, globulin fraction, albumin/globulin ratio, calcium, inorganic phosphorus, sodium, potassium, and chloride. The clinical variables were analyzed using ANOVA, with Tukey’s multiple range tests for pair-wise comparisons between the seven groups.

RNA was isolated from the frozen liver powder aliquots in batches of twelve samples at a time. Qiagen RNeasy mini kits (Qiagen, Chatsworth, CA, USA) were used for RNA extraction following the standard Qiagen protocol. Affymetrix GeneChip^®^ Rat Genome 230 v 2.0 arrays (Affymetrix, Inc., Santa Clara, CA) were used for DNA microarray analysis. A total of more than 28,000 well-substantiated rat gene probes was spotted on the chip. The built-in quality control was used to assess the labeling and hybridization performance of each DNA microarray and all were within the recommended ranges. Protocols from Affymetrix were followed to process the microarray chips. Twelve GeneChips were processed in a batch. An Affymetrix GeneChip^®^ Instrument System, which includes workstation, fluidics station, hybridization oven, and scanner, was used for the microarray processing (hybridization, washing, staining and scanning). The resulting images were analyzed by GCOS software to generate .cel files, which contain the raw intensity values of each probe. PLIER was used to convert the probe-level intensity data into normalized probeset-level intensity data using Affymetrix Gene Expression Console Software. The quality of each array was evaluated using Genedata Expressionist Refiner Array software.

### 3.2. Marker Identification and Categorization

The significance testing approach involved fitting a linear dose-response function to the dose-response data for each gene. For each drug, the slope of a linear dose-response model was estimated and tested for significance of the drug effect. The two estimated slopes were then compared to test for differences between the two drugs and determine the direction of the drug effects. FDR was applied to adjust multiple comparisons. The level of significance was set at an adjusted *p*-value = 0.05. For each comparison, the p-values were calculated using the permutation test from the normalized data. Candidate biomarker genes were selected according to the classification scheme described in Section 2.1.

The candidate biomarker genes identified were further evaluated using the self-organizing map (SOM) [23]. SOM is an unsupervised learning method that combines the features of dimension reduction in multi-dimensional scaling and non-hierarchical clustering. SOM divides the candidate marker genes into (k1 × k2) clusters; with each cluster representing a set of genes that have similar dose-response profiles for both drugs. The category of each of the (k1 × k2) clusters is then determined using the binomial likelihood probability. Since SOM is an unsupervised learning method, the analysis provides an independent validation of the categorization based on the significance testing approach.

### 3.3. Gene Set Enrichment Analysis (Pathway Analysis)

Gene set enrichment analysis (GESA) is a statistical procedure to determine a functionally related set of genes that are expressed differentially in different experimental groups [24]. The common approach to GESA is to generate a list of differentially expressed genes, and then compare the number of genes in the list with the total number of genes in the set to determine the significance for an over-representation in the gene set [25]. This approach has several short-comings [26]. First, the division of genes into differential and non-differential expression groups is arbitrary, and the genes in the non-differential expression list are discarded, regardless of their p-values. Second, the approach simply counts the number of genes in the list; the order of genes is not taken into consideration. Third, the Fisher's exact test assumes the genes are independent, and it does not take the correlation structure in the gene class into consideration. Because of these limitations, we used a two-sided MANOVA method [27] to identify which pathways were expressed differently among the vehicle and high-dosed groups of the two drugs, since the MANOVA method was found to perform better than the existing gene set analysis methods.

Pathway information, based on Kyoto Encyclopedia of Genes and Genomes [28], was generated from the Affymetrix GeneChip^®^ Rat Genome 230 v 2.0 chip by using ArrayTrack toxicogenomics software [29], and 193 pathways were identified. The pathways containing at least one candidate marker identified from the significance test were analyzed using the MANOVA.

## 4. Conclusions

The existence of relatively small, unidentified, sensitive subpopulations may explain the occurrence of idiosyncratic liver injury that is found only after approved drugs have been prescribed for large numbers of patients. Pre-clinical evaluations of investigational drugs typically use homogeneous populations of healthy animals. Even if the population response to a drug does not indicate any concerns of DILI, individual animals that are considered “outliers” may exhibit signs of DILI. These outlying responses are often difficult to interpret since they may occur in only a single animal out of many hundreds spread across multiple studies. However, these outliers may represent animals that have a true unique sensitivity to the drug. Identification of the susceptibility factors in these animals would greatly enhance the ability to identify idiosyncratic drugs prior to human exposure.

When considering clinical testing of new drugs, a “*hidden* sensitive” subpopulation that is very small relative to the overall human population may result in signs of potential liver toxicity being missed due to relatively small sample sizes. Clinical trials in humans also involve relatively homogeneous participants, especially during the earlier clinical trial phases. In order to be able to protect sensitive subpopulations, methods that can identify the small subset of idiosyncratic patients must be developed.

Commonly used diagnostic markers for hepatic injury include ALT, AST, TBIL, and ALP [2,30]. For example, an elevation of the ALT greater than 3 times the upper limit of normal is considered be a signal of liver toxicity. Idiosyncratic subpopulations that are not identifiable in conventional pre-clinical or clinical testing might be attributed to small sample sizes and/or small elevations in clinical chemistry markers. In Table 1, the difference between the vehicle and each of the tolcapone groups was not significant. Significant results were observed, however, between the high dose group of tolcapone and the low dose group of entacapone for ALT, AST, and TBIL. These results led to the identification of two susceptible animals. However, a typical pre-clinical experiment only involves a control group and a single drug, with one or more dose groups. By considering a pair of compounds, as in the LTBS experiment, the responses between the two compounds can be compared.

Several potential factors that may contribute to DILI include age, sex, drug-specific factors, environmental factors, and genetic variability. In particular, genetic variability is considered to be a major factor [2]. The pharmaceutical industry and research institutes have expended much effort to explore applications of genomics, proteomics, and metabolomics to develop pre-clinical biomarkers for detecting hepatic injury (e.g., [31,32]). One approach has been to use panels of drugs and chemicals to identify patterns of changes in gene expression at sub-toxic exposures of the drugs/chemicals that might be predictive of hepatotoxicity [33–35]. The LTBS was designed to study five sets of related pairs of drugs, with entacapone and tolcapone being the first pair of the five. For each pair of drugs, the chemical structures and targets are similar so that biochemical responses of toxicity may be revealed among the many biochemical changes induced by each pair of compounds. If common adversely affected molecular components or pathways can be found among the five pairs, biomarkers for predicting idiosyncratic hepatotoxicity may be developed.

## Figures and Tables

**Table 1:** Means and standard errors of the clinical chemistry variables: Alanine aminotransferase (ALT), Alkaline phosphatase (ALP), Total bile acids (TBA), Creatinine (CREA), Blood urea nitrogen (BUN), Aspartate aminotransferase (AST), Sorbitol dehydrogenase (SDH), Albumin (ALB), Total protein (TP), Total bilirubin (TBIL), Lactate dehydrogenase (LDH), 5′-Nucleotidase (5′-NT), Glutamate dehydrogenase (GLDH). The superscript indicates a significance difference (*p* < −0.05) between a pairwise comparison from the Tukey’s multiple range test. The ANOVA *p*-values are shown.

Clinical Chemistry Parameter	ANOVA *p*-Values	Vehicle	Entacapone Low	Entacapone Medium	Entacapone High	Tolcapone Low	Tolcapone Medium	Tolcapone High
ALT^a^,^b^,^c^	0.023	73.75 (3.12)	69.17 (1.86)	72.17 (3.14)	76.25 (3.98)	70.08 (2.60)	69.75 (2.01)	87.67 (7.90)
ALP	0.580	290.75 (22.68)	342.75 (23.98)	317.75 (22.19)	319.50 (21.83)	297.25 (15.88)	309.83 (13.38)	335.33 (25.56)
TBA	0.376	15.95 (4.39)	14.65 (4.07)	8.09 (1.31)	13.05 (3.37)	9.60 (3.72)	7.81 (1.84)	9.37 (1.82)
CREA	0.753	0.65 (0.03)	0.73 (0.07)	0.68 (0.02)	0.71 (0.03)	0.68 (0.03)	0.71 (0.02)	0.67 (0.03)
BUN^d^	0.040	14.93 (0.35)	16.68 (0.54)	17.65 (0.88)	19.03 (1.67)	16.61 (0.43)	16.97 (0.53)	17.53 (0.47)
AST^a^	0.040	110.08 (8.28)	95.25 (5.14)	98.33 (4.27)	97.33 (3.77)	100.75 (4.70)	106.75 (6.40)	122.42 (9.12)
SDH	0.683	41.85 (5.34)	46.98 (2.51)	48.79 (3.69)	49.44 (3.67)	47.96 (4.66)	51.61 (4.60)	52.53 (5.06)
ALB	0.406	4.58 (0.06)	4.54 (0.10)	4.83 (0.07)	4.63 (0.11)	4.57 (0.10)	4.60 (0.09)	4.66 (0.12)
TP	0.752	6.13 (0.07)	6.18 (0.08)	6.21 (0.07)	6.22 (0.10)	6.12 (0.05)	6.19 (0.08)	6.28 (0.06)
TBIL^e^	0.048	0.14 (0.01)	0.25 (0.06)	0.14 (0.01)	0.15 (0.02)	0.13 (0.01)	0.15 (0.02)	0.18 (0.03)
LDH	0.063	381.83 (36.74)	370.75 (33.14)	368.00 (32.52)	377.67 (33.05)	341.50 (28.25)	462.83 (45.11)	491.50 (53.18)
5′-NT	0.184	32.93 (1.78)	38.79 (2.17)	33.17 (1.52)	32.76 (2.04)	31.71 (2.18)	31.48 (1.20)	33.96 (2.74)
GLDH	0.684	11.37 (1.55)	12.27 (2.41)	12.15 (2.67)	11.56 (1.65)	11.91 (1.81)	15.67 (2.66)	15.49 (2.82)

ALT-

a: significance between Tolcapone high dose and Entacapone low dose;

b: significance between Tolcapone high dose and Tolcapone low dose;

c: significance between Tolcapone high dose and Tolcapone medium dose.

BUN-

d: significance between Entacapone high dose and vehicle.

AST-

a: significance between Tolcapone high dose and Entacapone low dose.

TBIL-

e: significance between Tolcapone low dose and Entacapone low dose.

**Table 2:** The number of classified markers identified by the significance testing approach and the SOM validated for the 4 expanded and 4 primary categories. The concordance rate, average sensitivity (range), and average specificity (range) for each category are shown.

Method	Total	Marker Category			
		t (T)	e (E)	c (C)	d (D)
Significance Testing	695 (238)	223 (120)	319 (32)	93 (81)	50 (5)
SOM	472 (138)	118 (67)	245 (22)	77 (46)	32 (3)
Concordance rate	68% (58%)	53% (56%)	77% (69%)	83% (57%)	64% (60%)
Sensitivity (range)	0.71 (0.33–0.92)	0.72 (0.67–0.92)	0.71 (0.33–0.92)	0.71 (0.33–0.92)	0.74 (0.67–0.92)
Specificity (range)	0.69 (0.33–1.00)	0.70 (0.67–0.92)	0.69 (0.42–0.92)	0.69 (0.33–0.92)	0.73 (0.50–1.00)

**Table 3:** Tolcapone-specific pathways. These pathways consist of only *T* and/or *t* (indicated by an ^*^) category genes among the 695 genes identified. The table shows the pathway name, the number of genes in the pathway, the gene name(s) in the pathway, and the *p*-value of gene set analysis.

Gene Set	Number of Genes	Categorized Genes	*p*-Value
Galactose metabolism	16	Gck	<0.0001
Cysteine metabolism	16	Sult1c2	0.003
Aminoacyl-tRNA biosynthesis	16	Vars2	0.009
Wnt signaling pathway	112	Sox17 Dkk2 ^*^	0.014
Taste transduction	29	Gnat3 ^*^ Tas2r114	0.046
C21-Steroid hormone metabolism	10	Cyp21a1	0.1250
Chondroitin sulfate biosynthesis	14	B3gat2 Chst12	0.1260
Antigen processing and presentation	58	RT1-Aw2	0.163
